# AGE DIFFERENCES IN DECISION SUPPORT NETWORK SIZE: A STUDY OF TOTAL JOINT REPLACEMENT CANDIDATES

**DOI:** 10.1093/geroni/igae098.4113

**Published:** 2024-12-31

**Authors:** Tess Wild, Catherine Riffin, Corinna Löckenhoff

**Affiliations:** Cornell University, Ithaca, New York, United States; Weill Cornell Medicine, New York, New York, United States; Cornell University, Ithaca, New York, United States

## Abstract

Arthritis, which affects tens of millions of middle-aged and older adults, is one of the most common causes of activity limitation, disability, and chronic pain. Despite evidence that total joint replacement (TJR) can improve quality of life and physical functioning in arthritis patients, many eligible TJR candidates hesitate to pursue surgical intervention, and this reluctance is higher among older patients. Research shows that willingness to undergo TJR is influenced by a variety of psychosocial factors. Social relationships in particular can be an important source of support and information for patients when considering surgery. It is well documented that people’s social networks tend to be smaller and more close-knit as they age, and recent research suggests that decision support networks for hypothetical healthcare decisions show the same trend. The present study examined whether this pattern extends to realistic decisions about TJR. Using a name-elicitation approach, we collected information on the size of patients’ decision support networks (Msize = 3.30; rangesize = 0-8) in a sample of potential TJR candidates (N = 98; Mage = 67.88; rangeage = 40-93 years). As predicted, linear regression revealed that, compared to younger potential TJR candidates, older potential TJR candidates identified fewer members in their decision support networks (B= -0.033; p =.048). Additional analyses examined the role of potential covariates including demographics (e.g., gender, marital status), health (e.g., pain, functional status), and psychosocial variables (e.g., personality, health literacy). Theoretical implications and ramifications for clinical practice are discussed.

